# Mitochondrial DNA, chloroplast DNA and the origins of development in eukaryotic organisms

**DOI:** 10.1186/1745-6150-5-42

**Published:** 2010-06-29

**Authors:** Arnold J Bendich

**Affiliations:** 1 Department of Biology, University of Washington, Seattle, WA 98195-5325, USA

## Abstract

**Background:**

Several proposals have been made to explain the rise of multicellular life forms. An internal environment can be created and controlled, germ cells can be protected in novel structures, and increased organismal size allows a "division of labor" among cell types. These proposals describe advantages of multicellular versus unicellular organisms at levels of organization at or above the individual cell. I focus on a subsequent phase of evolution, when multicellular organisms initiated the process of development that later became the more complex embryonic development found in animals and plants. The advantage here is realized at the level of the mitochondrion and chloroplast.

**Hypothesis:**

The extreme instability of DNA in mitochondria and chloroplasts has not been widely appreciated even though it was first reported four decades ago. Here, I show that the evolutionary success of multicellular animals and plants can be traced to the protection of organellar DNA. Three stages are envisioned. *Sequestration *allowed mitochondria and chloroplasts to be placed in "quiet" germ line cells so that their DNA is not exposed to the oxidative stress produced by these organelles in "active" somatic cells. This advantage then provided *Opportunity*, a period of time during which novel processes arose for signaling within and between cells and (in animals) for cell-cell recognition molecules to evolve. *Development *then led to the enormous diversity of animals and plants.

**Implications:**

The potency of a somatic stem cell is its potential to generate cell types other than itself, and this is a systems property. One of the biochemical properties required for stemness to emerge from a population of cells might be the metabolic quiescence that protects organellar DNA from oxidative stress.

**Reviewers:**

This article was reviewed by John Logsdon, Arcady Mushegian, and Patrick Forterre.

## Background

In order for its lineage to persist, an organism must transmit its genetic information to the next generation and it uses the chromosome as the vehicle of inheritance. Since DNA is continuously exposed to damaging agents, however, the problem arises as to how to transmit undamaged chromosomal DNA to the progeny. For the nucleus of a eukaryotic cell, the DNA repair problem is relatively mild because damage is relatively low. The DNA repair problem is more severe for mitochondria and chloroplasts because the energy demands of the cell are met largely by electron transport in these organelles, which generates reactive oxygen species (ROS) as unavoidable by-products. The ROS can lead to oxidative stress and on-site DNA damage. In a unicellular organism, DNA repair is the only way to maintain pristine chromosomal DNA in the cytoplasmic organelles. On the other hand, DNA damage could be *avoided *if cells specialized for reproduction were available in which oxidative stress is suppressed: a multicellular organism containing somatic and germ line cells.

A somatic cell needs to be metabolically active. As conditions change during development or in an adult tissue, energy needs will change. There will be episodes of increasing and decreasing demand for ATP from mitochondria and chloroplasts, including sudden changes in growth rate, temperature, light and water availability, and pathogen and toxin challenge. An active cell is one responsive to such change and will unavoidably experience episodes of increasing and decreasing ROS and oxidative stress. A quiet cell is not metabolically equipped for rapid change and thus experiences minimal oxidative stress, precisely the conditions for protecting germ line DNA from damage. The segregation of active and quiet metabolic states into separate cell types created an adaptive advantage that then provided the time required for the evolutionary "experiments" that led to development in multicellular organisms (see Appendix 1). Cells that later will become gametes are kept quiet early in development. After the germ line organellar DNA is sequestered, somatic tissues and organs develop to the point at which reproduction is most likely to succeed. Then the gametes with their pristine organellar genomes are deployed.

The first multicellular organisms probably arose from preexisting unicellular forms. Subsequently, some multicellular organisms used development to create specialized parts in the adult. I am unaware of any previous analysis of the origins of development. Several proposals, however, have been made to explain the rise of multicelluar life forms [1-4]. An internal environment can be created and controlled, germ cells can be protected in novel structures, and increased organismal size allows a "division of labor" among cell types including germ cell protection. These proposals describe advantages of multicellular versus unicellular organisms at levels of organization at or above the individual cell, and the unicellular-to-multicellular phase of evolution preceded the phase addressed in my hypothesis. In some muliticellular eukaryotic lineages there are only two cell types, one of which is a reproductive spore or germling (slime molds or *Volvox*, for example; see Appendix 1). My hypothesis concerns those lineages in which a developmental process arose to sequester germ cells from somatic cells, leading to the advantages of organellar DNA protection and DNA repair cost savings. These advantages allowed the evolution of additional somatic cell types and more complicated organisms, some of which employed gastrulation and others with permanently attached cells did not.

## Presentation of the hypothesis

### Instability of DNA in mitochondria and chloroplasts

The DNA in both mitochondria and chloroplasts can be extremely unstable, as illustrated by the following examples. (i) The half-life of rat mitochondrial DNA (mtDNA), in days, is 6.7 for heart, 9.4 for liver, 10.4 for kidney, and 31 for brain [5]. (ii) In the single-celled alga *Euglena*, the half-lives for chloroplast DNA (cpDNA) and mtDNA are 1.6 and 1.8 cell doublings, respectively, but nuclear DNA is so stable that turnover could not be detected [6,7]. (iii) Two days after sowing mung bean seeds, the mtDNA in dark-grown seedlings turns over entirely in 24 hours [8]. (iv) The half-life of mtDNA in yeast is ~4 hours (for a mutant defective in the mtDNA polymerase gamma) [9]. (v) Light triggers the degradation of DNA in maize chloroplasts [10]. Four hours after exposing 10-day-old dark-grown seedlings to light, the leaf begins to green, and the average DNA content per chloroplast decreases to 54% by hour 6 and 9% by hour 24 (unpublished results from my laboratory). (vi) During 6 stages of development of maize leaf tissue, the size and structural integrity of cpDNA decreases progressively from branched molecules of multigenomic size in the basal meristem of seedlings to fragments of subgenomic size in adult plants, as observed in moving pictures of individual ethidium-stained DNA molecules [10]. A similar degradative progression of individual cpDNA molecules is observed during leaf development for tobacco and the legume *Medicago truncatula *[11] and Arabidopsis [12]. (vii) In fully expanded leaves of adult plants of Arabidopsis [12,13] and maize [10], more than half the chloroplasts contain no detectable DNA.

How can we explain this remarkable instability of organellar DNA? I suggest that the ROS generated during electron transport that accompanies oxidative phosphorylation and photosynthesis leads to oxidative stress and extensive damage to the DNA. For *Euglena*, repair of the mtDNA and cpDNA is the only option because it is a unicellular organism. For dark-grown mung bean seedlings, repair again is the only option for mtDNA since respiration must provide the energy for this aerobic organism. The mtDNA is so extensively damaged that it turns over completely in one day. For a light-grown plant, however, there is another option. If some of the organellar DNA can be sequestered in quiescent germ line cells, the highly damaged organellar DNA in somatic cells can be left unrepaired; it is eventually degraded and its nucleotides are recycled for their nutritive value [14]. Similarly, oxidatively damaged mtDNA in active somatic cells can either be repaired or abandoned, as long as undamaged mtDNA is retained in quiet germ line cells. For the mesozoan *Dicyema japonicum*, mtDNA is retained in "stem" mitochondria of germ cells, but mtDNA is undetectable in most somatic cells of mature larvae and adults, a result of either dilution without replication [15] or, I suggest, abandonment and degradation of mtDNA.

### DNA damage and repair in mitochondria and chloroplasts

From an evolutionary perspective, the only objective for an organism is to replicate its DNA and pass it on to the next generation. Unintended alterations in chromosomal DNA molecules can arise in various ways, including DNA polymerase errors and changes to the DNA template from internal (ROS, for example) and external (radiation, for example) sources. I will consider only internal sources because these can be modulated during development. Changes in DNA can be perceived and acted upon as needed during development.

Changes in DNA can occur as nucleotide alterations, insertions/deletions, inversions, and DNA strand breaks. Those lesions recognized as "damage" can be either repaired or removed by degrading the DNA [16]. Most information on the repair of mtDNA comes from yeast and somatic cells of mammals [17-21], whereas very little is known about mtDNA repair in plants or about cpDNA repair [22-25]. A detected change in mtDNA is the result of both the rate of damage and the efficiency of correcting the damage. The power of genetics can sometimes be used to study each of these parameters separately in yeast.

Overall, two conclusions seem generally supported. First, most DNA damage in mitochondria is due to oxidative damage, as may be expected for the site of respiration, and base excision repair (BER) is the main way to rectify oxidative damage [18,26,27]. If BER fails, human mtDNA molecules containing the damage are usually degraded and base substitution (point mutation) is thus avoided [28]. Degradation of damaged DNA molecules to avoid mutation is feasible for the high-genome-copy cytoplasmic organelles, but not for the diploid nucleus. Such degradation would mask a higher rate of damage in the organelles than in the nucleus. The second conclusion is that the capacity to repair stress-induced DNA damage is lower in mitochondria than the nucleus, because mitochondria are the principal site of ROS production, employ fewer repair processes than do nuclei, or lack protective histones on their mtDNA molecules [17,18,27,29-31]. Damage to organellar DNA is indicated by a rapidly increasing mutation rate (point mutations per kb of mtDNA) as mouse tissues age [26], an accumulation of mtDNA deletions with age in humans, monkeys, and rodents [17,32], and a decline in structural integrity of cpDNA molecules as leaves develop (discussed above). Thus, it would be advantageous to shelter organellar DNA before tissues mature in the adult.

### Quiet and active metabolism

How might a cell achieve quiet metabolism in order to protect its organellar DNA? Most of our information on the regulation of mitochondrial biochemistry comes from yeast (where embryogenesis does not occur) and mammals (where embryogenesis has been intensively investigated). When grown under nutrient-limited conditions, yeast cells alternate between glycolytic and respiratory metabolism; they replicate DNA only during glycolysis, thereby avoiding oxidative stress and DNA damage (only nuclear DNA was analyzed) [33]. In early mammalian embryogenesis, "reducing equivalents (or electron donors) and metabolic intermediates formed during mitochondrial oxidative reactions are diverted from ATP production and redistributed to the cell to supply biosynthetic pathways and regenerate antioxidant defence (a process called mitochondrial anaplerosis)" [34]. I suggest that anaplerosis may be used to diminish oxidative stress and maintain metabolic quiescence in germ line cells of any organism that undergoes development.

The quiescent cells may be fated to become germ cells by maternally inherited determinants before, or immediately following, fertilization ("preformation" or "primordial germ cells"), as in amphibians, for example [35]. But in most animals, including mammals, germ cells are not observed until later in development and arise as a result of inductive signals from surrounding tissues ("epigenesis"). Similarly, plant germ cells are derived epigenetically from quiet cells originating in the shoot apical meristem [36]. Thus embryogenesis allows germ cells to arise only from metabolically quiescent cells, either early (preformation) or later (epigenesis) in development.

Establishing a quiet cell may not be mechanistically complex. The rapid proliferation of mitochondria in yeast cells can be initiated by TOR (Target Of Rapamycin) signaling [37,38]. A single nuclear DNA regulatory element has been proposed to control energy genes in both the nucleus and mitochondrion [39], and an analogous coordination has been proposed for regulating energy production by chloroplasts [40]. The ground or default state may be energetically quiet mitochondria that are simply not activated in germ line cells. The colorless proplastids in angiosperm meristems are in a ground state and would not experience the cpDNA-damaging conditions created by photosynthesis in green chloroplasts. Thus, merely not turning "on" organellar development may passively reduce the DNA repair burden.

**Figure 1:**
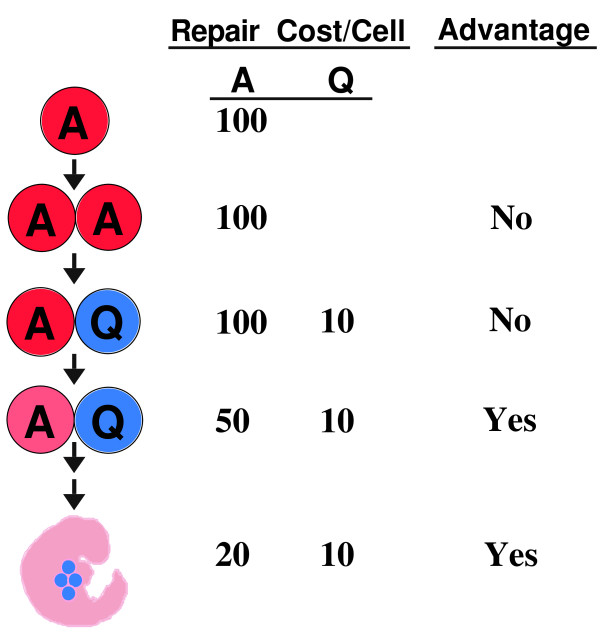
**DNA repair cost during the evolution of development**. The single cell of a protist is metabolically active (A) and generates ROS and oxidative stress during mitochondrial respiration. Oxidative stress leads to mtDNA damage that must be fully repaired at a cost of 100. An organism containing two A cells gains no selective advantage. Conversion of one A cell to a metabolically quiescent (Q) cell reduces the damage load and repair cost, but this two-celled organism gains no advantage. If the Q cell acts as a germ cell, the organism gains an advantage by lowering the repair cost in its A cell, even though some A-cell mtDNA remains unrepaired and may be degraded. At this point, many evolutionary "experiments" can occur with multicellular embryos, and those allowing further reduction in cost-per-A-cell gain a large advantage. For example, the multi-copy nature of mtDNA in humans provides sufficient mtDNA of "good enough" quality to reach sexual maturity, after which degenerating mitochondrial function leads to aging symptoms. An analogous scenario can be made for cpDNA.

In summary, organellar DNAs could be sequestered into a germ cell lineage where oxidative stress was avoided. The DNA repair burden would also be reduced in somatic cells because some damage to organellar DNA could be tolerated. The benefit of reduced DNA repair cost would increase as the number of somatic cells increases (Fig. 1).

### The hypothesis

The transition from unicellular to multicellar organisms has been analyzed previously [1-4]. My hypothesis concerns a subsequent phase of evolution, when multicellular organisms initiated the process of development that led to organisms with more than one type of somatic cell.

Three stages are envisioned. The protection from oxidative stress afforded by *Sequestration *of organellar DNA conferred a selective advantage that allowed these organisms to persist for a time period during which evolutionary "experiments" in the *Opportunity *stage provided the next advances toward more complicated eukaryotes. One such advance was novel signaling within and between cells. Although excess ROS can be deleterious, low levels of ROS are used as signaling molecules during normal development in animals and plants [41-43], as are reactive nitrogen species (RNS) such as nitric oxide (NO) [44]. Both ROS and RNS can be produced at cytochrome c oxidase of the electron transport chain in mitochondria, depending on the oxygen concentration. Superoxide and NO can react to form peroxynitrite (another RNS), leading to sophisticated hypoxic signaling between mitochondrion and nucleus and to intercellular signaling [45]. The potential to increase biological complexity by cell-to-cell signaling would be greater in organisms with several types of somatic cells than in organisms with only one type of somatic cell (see Appendix 1). Another advance during the *Opportunity *stage was the creation of a repertoire of cell adhesion molecules to orchestrate cell movements during gastrulation in animals [46]. Advances such as these provided the biochemical circuitry used in the final stage, *Development*, to produce animals and plants.

Although my hypothesis extends only to the point at which development first appears in an evolutionary lineage, two comments can be made concerning the forms of development that appeared later. First, the developmental process became more complicated in some lineages (gastrulation in mammals, for example) than others (no gastrulation in mesozoans [47] or plants where cells are permanently attached by rigid cell walls). Second, the "Cambrian explosion" of animal bodyplans [48] may reflect the success of the protection-by-sequestration of organellar DNA.

There are other eukaryotic lineages in which morphologically complex organisms may have arisen by this three-stage process. Within the Phaeophyceae (brown algae) and Rhodophyta (red algae), a maturation process leading to an adult with several cell types occurs, but no structure that resembles the type of embryo found in animals and plants is observed [49]. Mushrooms are the morphologically most complex fungi [50]. The mature fruiting body (the "mushroom") is essentially a swollen version of the much smaller "embryo" without a great increase in the number of cells, unlike the embryo-adult relationship in animals and plants. Furthermore, it is uncertain whether there is only one or more than one type of somatic cell, and except for the spore-forming basidia, all cells are totipotent [50]. Phylogenetic analysis suggests that animals, plants, brown algae, red algae, and fungi arose independently, with most eukaryotic diversity represented by microbial protists (protozoa and algae) [51,52]. Thus the transition from one to more than one somatic cell type may have occurred several times, although it is also possible that this transition occurred only once and the process of development was lost in protists. These various issues need to be addressed before deciding whether my hypothesis for the origin of development applies beyond animals and plants.

## Testing the hypothesis

The protection-by-sequestration hypothesis specifies that ROS production will be much lower in germ line cells than somatic cells of animals and plants. Certain fluorescent dyes can be used to quantify ROS levels in cells [53]. To overcome the difficulty in obtaining adequate numbers of germ line cells from mammals, ROS assays have been conducted with cultured cells. However, metabolic quiescence may be lost during cultivation *in vitro *[38]. Plants like maize are well-suited for such tests because of the relative ease with which meristematic tissue can be obtained directly from the plant [54]. Comparative ROS assays should also be conducted with non-angiospermous land plants and multicellular algae in the presence and absence of light.

DNA damage leads to mutation. The nucleotide sequence of organellar genomes should vary minimally among individual germ line cells, but greater variation among individual somatic cells would be expected because their active metabolism should increase the mutation rate. Single-cell DNA sequencing would provide data to test this prediction.

The ROS assay and single-cell organellar DNA sequencing could show whether an organism produces embryos with quiet organelles or whether development is non-embryogenic, as in fruiting bacteria and cellular slime molds (see Appendix 1). On the other hand, such analyses may prove inconclusive. In rhododendron, an evergreen plant, we found DNA in green chloroplasts from leaves that remained on the branch for each of the five years that leaves were produced on that branch (unpublished results from my laboratory). Embryonic development provides the organism with a way to reduce the cost of organellar DNA repair in somatic cells (Fig. 1), but that cost may be incurred in pursuit of an ecological niche, such as an evergreen lifestyle.

## Implications of the hypothesis

It has been suggested that one requirement for somatic stem cells in mammals is that they be at least bi-potent (able to produce at least two cell types other than themselves) [55]. Meristematic cells in the shoot apex of angiosperms would qualify as stem cells, using this bi-potency criterion, but multicellular slime molds and *Volvox *would not contain such stem cells (see Appendix 1). One of the biochemical properties required for stemness to emerge from a population of cells might be the metabolic quiescence that protects organellar DNA from oxidative stress.

Endosymbiosis led to mitochondria and chloroplasts and a great increase in cellular complexity. Development led to animals and plants and a great increase in organismal complexity. As proposed, the origin of development can be traced to protection of mtDNA and cpDNA. A seemingly ironic conclusion emerges: organismal complexity arose primarily because the minor fraction of genes located in cytoplasmic organelles could be protected from the by-products of their own activities. Another irony is that the advent of development has more to do with protecting the few organellar genes than the many nuclear genes.

## Competing interests

The author declares that he has no competing interests.

## Appendix 1. Multicellularity and embryonic development

Not all multicellular organisms experience embryonic development. A multicellular organism can result from the association of previously unattached individual cells (aggregative development) or by growth and division of a single cell [56]. In animals and plants, the progenitor cell can be a zygote or a totipotent stem cell, and embryonic development proceeds as groups of cells differentiate and form increasingly specialized structures that lead to the more complex parts of the adult. A macroscopic fruiting structure containing two cell types, stalk and spore, can be produced by aggregative development from prokaryotic cells (fruiting myxobacteria [57]) or eukaryotic protist cells (cellular slime molds *Dictyostelium *and *Acrasis *[56]). In aggregative development, cells of different genotype can co-aggregate, whereas in embryonic development all cells are clonal. *Volvox carteri *is a chlorophytic (green) alga that produces an adult containing only two cell types: > 2000 somatic cells and ~16 gonidia, asexual reproductive cells [58]. Each gonidium initiates cleavage divisions to produce an "embryo" that contains all the cells that will be present in an adult of the next generation. For animals and plants, the progression from single cell to adult clearly includes an embryo. Whether *V. carteri *experiences embryonic development may be a matter of semantics.

The spore of bacteria and slime molds and the gonidium are specialized reproductive cells produced *after *DNA damage has been repaired, and thus the benefit of protection-by-damage-avoidance is not realized. This may be an important distinction between embryogenesis in animals and plants and the type of multicellular development in the other groups. The damage-avoidance benefit may have allowed animals and plants to produce many cell types, not just the two (somatic and spore/gonidium) found in the other groups.

## Reviewers' comments

### Reviewer's report 1

Patrick Forterre, Unité de Biologie du Gène chez les Extrêmophiles, Institut Pasteur, Paris, France

The author addressed each of my detailed comments and I find the revised version acceptable for publication.

### Reviewer's report 2

John M. Logsdon, Jr., Department of Biology, University of Iowa, Iowa City, IA 52242, USA

This manuscript puts forward a provocative idea to connect the genesis of embryonic development with the protection of organellar genome integrity. The hypothesis is premised strongly on observations that organellar genomes are highly unstable in most somatic tissues of animals and plants, with DNA experiencing considerable degradation from oxidative metabolism. In such (embryonic) organisms, the segregation of the soma and germ line is posited to provide a protection for organellar DNA in the metabolically quiescent germ cells. This organelle sequestration is then hypothesized to be the initial step eventually leading to what we now recognize as embryogenesis.

Although the author provides an interesting scenario to connect organelles, germ lines, and embryonic development, I think that an alternative evolutionary sequence could also underpin the origin of embryogenesis: that organelle sequestration and its effect on reducing the mutational burden of organelle genomes was a (perhaps necessary) consequence of embryogenesis. In other words, what is cause and what is consequence?

### Author's response

My hypothesis (let us call it the forward scenario) is that the advent of development provided a means (organelle sequestration) to avoid the repair cost necessary to reduce the mutational burden. As such, this sequence of events is clearly beneficial, and that benefit can be seen in the downward flow of events in Figure 1. The reverse scenario (organelle sequestration was "perhaps necessary" to reduce the mutational burden) implies that the advent of development *created *a mutational burden, which then had to be alleviated by a subsequent invention, sequestration. The reverse scenario begins with a detrimental event (embryogenesis somehow increased the mutational burden--but how and why should this happen?) that was counteracted by the subsequent sequestration of organelles. Since the benefit in the reverse scenario is not apparent, I do not consider this scenario in the text.

### Reviewer's comment

It is unclear if this hypothesis will find resonance in the organelle and/or embryogenesis literature. However, the author provides some suggested tests of its implications. As evolutionary "origins" questions are often very difficult to solve, suggestions like these may be welcome in the marketplace of ideas.

### Reviewer's report 3

Arcady Mushegian, Department of Binformatics, Stowers Institute for Medical Research, Kansas City, MO 64110, USA

The hypothesis put forward by Bendich in this manuscript states that:

(a) the energy-producing stations of an eukaryotic cell, i.e., mitochondria and chloroplasts, do their job at a peril for the integrity of their own DNA, because of the ROS and their derivatives that are damaging to DNA;

(b) multicellularity, and later embryonic development, have originated as the adaptations to damage and loss of organellar DNA, by sequestration in metabolically quiet subset of cells.

### Author's response

The assertion in (b) is not correct. My hypothesis explains the adaptive significance of development, but not of multicellularity. The adaptive significance of multicellularity was analyzed in detail by others, cited in my refs [1-4]. My hypothesis begins with existing multicellular organisms and concerns the subsequent advent of the process of development.

### Reviewer's comment

Part (a) seems to be supported by biochemical and cytological evidence lovingly collected by the author from his own studied and from the literature. These data are very interesting. As for Part (b), I am less enthusiastic. Indeed, the advent of multicellularity and the origin of evolutionary development are two different events that may require different explanations. Moreover, even if relative protection from DNA damage is a factor in the evolution of multicellular organisms, it could be an additional benefit, not the main force behind the emergence of either multicellularity or embryogenesis. Finally, there are many other suggestions in the literature concerning the origin of multicellularity and embryogenesis. As any other hypothesis, this one has to be evaluated both on its own merits and in comparison with other hypotheses.

### Author's response

The main criticism in this comment is that these are two different events that may require different explanations. I agree completely with this notion, and the text now clearly distinguishes multicelluarity from development, both in time of origin and proposed benefit. Surprisingly, despite an extensive effort, I could find no explanation in the literature for why or how development originated and no proposal for a benefit provided by multicellularity-plus-development versus multicellularity. Although it is possible that protection from DNA damage is "not the main force behind the emergence of either multicellularity or embryogenesis", there are simply no other explanations I could find in the literature or in discussions with colleagues.

### Reviewer's comment

Specifically, I think three opportunities are missed in this proposal:

1. Discuss the hypothesis vis-a-vis those that place more emphasis on stochastic population effects, most importantly, the line of argument by M. Lynch.

2. Give considerable attention to unicellular eukaryotes, to fungi that may adopt either unicellular or differentiated lifestyle, and to colonial eukaryotes - can author's hypothesis be tested by examining these forms that have organelles but lack defined germline or embryogenesis?

3. Examine the genomic evidence: under the author's hypothesis, as a first approximation, should the number of genes (or perhaps the number of gene products per cell) that encode the organellar repair machinery scale slower than the number of genes in the genome, or at least lower than the number of nuclear repair genes?

### Author's response

1. As I state above, I could find no other competing hypotheses concerning the origin of development. I sent an email to Michael Lynch in which I asked if he "could direct me to an article by you (or anyone else) that addresses the benefits of embryonic development that goes beyond the transition from unicell-to-multicell", but I received no response. In searching Lynch's articles, the one that seemed most relevant was "The frailty of adaptive hypotheses for the origins of organismal complexity" [PNAS 104:8597-604, 2007]. I have no disagreement with the arguments in this article. However, Lynch's arguments and conclusions are not relevant to my hypothesis.

2. Unicellular and colonial eukaryotes are already discussed in some detail in **Appendix 1 **and again in the **Testing the hypothesis **section. In ***The hypothesis ***section, I consider groups other than animals and plants, including protists, and conclude that we presently have insufficient information to extend the hypothesis beyond animals and plants.

3. I have no insight or predictions concerning the scaling of the number of genes or gene products for the DNA repair machinery between organelle and nucleus. Given the brevity with which this issue is described, I do not know how to respond.
